# Superoxide Initiates the Hyphal Differentiation to Microsclerotia Formation of *Macrophomina phaseolina*

**DOI:** 10.1128/spectrum.02084-21

**Published:** 2022-01-26

**Authors:** Hsien-Hao Liu, Cheng-Chun Huang, Ying-Hong Lin, Min-Nan Tseng, Hao-Xun Chang

**Affiliations:** a Department of Plant Pathology and Microbiology, National Taiwan University, Taipei City, Taiwan; b Department of Plant Medicine, National Pingtung University of Science and Technology, Pingtung, Taiwan; c Kaohsiung District Agricultural Research and Extension Station, Council of Agriculture, Pingtung, Taiwan; University of Molise

**Keywords:** hydrogen peroxide (H_2_O_2_), *Macrophomina phaseolina*, microsclerotia, reactive oxygen species (ROS), superoxide (O_2_^−^)

## Abstract

The infection of *Macrophomina phaseolina* often results in a grayish appearance with numerous survival structures, microsclerotia, on the plant surface. Past works have studied the development of fungal survival structures, sclerotia and microsclerotia, in the Leotiomycetes and Sordariomycetes. However, M. phaseolina belongs to the Dothideomycetes, and it remains unclear whether the mechanism of microsclerotia formation remains conserved among these phylogenetic clades. This study applied RNA-sequencing (RNA-Seq) to profile gene expressions at four stages of microsclerotia formation, and the results suggested that reactive oxygen species (ROS)-related functions were significantly different between the microsclerotia stages and the hyphal stage. Microsclerotia formation was reduced in the plates amended with antioxidants such as ascorbic acid, dithiothreitol (DTT), and glutathione. Surprisingly, DTT drastically scavenged H_2_O_2_, but the microsclerotia amount remained similar to the treatment of ascorbic acid and glutathione that both did not completely eliminate H_2_O_2_. This observation suggested the importance of ${\text{O}}_{2}^{-}$ over H_2_O_2_ in initiating microsclerotia formation. To further validate this hypothesis, the superoxide dismutase 1 (SOD1) inhibitor diethyldithiocarbamate trihydrate (DETC) and H_2_O_2_ were tested. The addition of DETC resulted in the accumulation of endogenous ${\text{O}}_{2}^{-}$ and more microsclerotia formation, but the treatment of H_2_O_2_ did not. The expression of SOD1 genes were also found to be upregulated in the hyphae to the microsclerotia stage, which suggested a higher endogenous ${\text{O}}_{2}^{-}$ stress presented in these stages. In summary, this study not only showed that the ROS stimulation remained conserved for initiating microsclerotia formation of M. phaseolina but also highlighted the importance of ${\text{O}}_{2}^{-}$ in initiating the hyphal differentiation to microsclerotia formation.

**IMPORTANCE** Reactive oxygen species (ROS) have been proposed as the key stimulus for sclerotia development by studying fungal systems such as Sclerotinia sclerotiorum, and the theory has been adapted for microsclerotia development in Verticillium dahliae and Nomuraea rileyi. While many studies agreed on the association between (micro)sclerotia development and the ROS pathway, which ROS type, superoxide (${\text{O}}_{2}^{-}$) or hydrogen peroxide (H_2_O_2_), plays a major role in initiating hyphal differentiation to the (micro)sclerotia formation remains controversial, and literature supporting either ${\text{O}}_{2}^{-}$ or H_2_O_2_ can be found. This study confirmed the association between ROS and microsclerotia formation for the charcoal rot fungus Macrophomina phaseolina. Moreover, the accumulation of ${\text{O}}_{2}^{-}$ but not H_2_O_2_ was found to induce higher density of microsclerotia. By integrating transcriptomic and phenotypic assays, this study presented the first conclusive case for M. phaseolina that ${\text{O}}_{2}^{-}$ is the main ROS stimulus in determining the amount of microsclerotia formation.

## INTRODUCTION

Macrophomina phaseolina is a plant-pathogenic fungus that causes charcoal rot on a broad range of crops, including monotcuts such corn and sorghum, as well as dicots such as azuki bean and soybean (1, 2). Yield losses caused by M. phaseolina on soybean were reported over 3 billion bushels (3), and charcoal rot was recognized as the second most important disease of soybean in a meta-analysis of 20 years of data (2). There is no complete resistance reported for charcoal rot, but some soybean varieties were found to exhibit moderate resistance (4). In addition, disease management may rely on the conventional practices such as appropriate irrigation and fungicide treatments (5–7). As M. phaseolina persists in debris and soils for years by forming the survival structure microsclerotia, advanced studies on the microsclerotia formation may provide novel insights to reduce the fungal survival structures in fields.

There are different types of sclerotia-like fungal structures, such as the true sclerotia, the pseudosclerotia, the small sclerotia, and the microsclerotia. All these structures were reported to have survival capability. Among these sclerotia-like fungal structures, the first description of sclerotia was named by De Bary in studying Sclerotinia sclerotiorum in 1886 (8). The earliest definition of a “sclerotium” can be tracked back to the documentation provided by Ainsworth and Bisby as “a firm, frequently rounded mass of hyphae, with or without the addition of host tissue or soil, normally having no spores in or on it” (9, 10). Later, the true sclerotium depicts a specialized fungal structure with a clear and melanized outer rind to separate the middle layer of cortex and the inner layer of medulla. The true sclerotia are multilayer structures that can be 2 to 8 mm on beans (Phaseolus vulgaris) and 12 cm or even larger on sunflowers (Harpalium Cass.) (11, 12). The composition of true sclerotia contains only hyphal mass without plant tissues or soils, and the true sclerotia are capable of forming apothecia to disseminate ascospores.

The second type of sclerotia was called “pseudosclerotia,” which describes the fungal survival structures with melanized and condensed mycelia, generally wrapped with plant tissues or soils (13). Fungal conidia may occur on the pseudosclerotia, which may produce apothecia to disseminate ascospores as the primary inoculum in the new season. A typical example will be the mummified survival structure of Monilia species on stone fruits (14). On the other hand, the third type of sclerotia was called “small sclerotia,” which was first described by R.E. Smith related to lettuce infection caused by S. sclerotiorum (15). However, the small sclerotia was conceptualized as sclerotia of 0.5 to 2 mm, with an emphasis on its ability of infecting plants using hyphae grown out from the small sclerotia instead of forming apothecia to disseminate ascospores (16). Based on the morphological and physiological differences, the isolate was reclassified the as a new species, Sclerotinia minor, which becomes the species type for producing small sclerotia (17).

Similar to the observation on the small sclerotia, the fourth term “microsclerotia” describes a tiny melanized and condensed survival structure of Verticillium species. Different from sclerotia and small sclerotia, the development of microsclerotia was proposed to begin with a single hypha. In the case of Verticillium dahliae, microsclerotia formation was initiated by lateral budding on a single hypha, which becomes a spherical compact microsclerotia with melanized and hyaline cells inside (18). The inner hyaline cells were proposed to be autolyzed cells during the differentiation, and these cells may not be viable for germination (19). In the case of M. phaseolina, microsclerotia formation was initiated by secondary branching hyphae wrapping on the primary hypha, which develops into the globose structure. Alternatively, the primary hyphae may proliferate and protrude to increase the cell mass for microsclerotia formation (20). Different from microsclerotia of V. dahliae, the inner cells of M. phaseolina microsclerotia were found to be all melanized with capability of germination (19).

Although histological studies have categorized fungal sclerotia into at least four types aforementioned, the initiation of sclerotia formation appears to be conserved based on the studies of Athelia rolfsii (previously known as *Sclerotium rolfsii*), S. minor, S. sclerotiorum, and Rhizoctonia solani. Using these fungal systems, the theory of reactive oxygen species (ROS)-induced sclerotia development was conceptualized (21). The theory was proposed when the concentration of malondialdehyde in the total phospholipids was measured to be higher in the center of a fungal colony, where more sclerotia formed. This observation suggested that the lipid peroxidation, an indicator of intracellular ROS stress, was associated with sclerotia formation of A. rolfsii (22). In addition, the oxidative stress comparison between sclerotia-forming and non-sclerotia-forming S. sclerotiorum strains revealed a 5-fold difference in reduced form over oxidized form of erythroascorbate (23). Subsequent studies applied antioxidants such as ascorbic acid to A. rolfsii (24), S. minor (25), S. sclerotiorum (23), and R. solani (26), and these studies consistently resulted in the reduction of sclerotia formation. The application of ROS scavengers such as benzoate and ethanol also resulted in the reduction of sclerotia formation (27). In addition, the measurement of hydrogen peroxide (H_2_O_2_) was found to be higher along with sclerotia development (28–30), and the addition of radical stimulator H_2_O_2_ increased cell proliferation and sclerotia formation in A. rolfsii and S. sclerotiorum (31). Collectively, these studies demonstrated a strong association between ROS production and sclerotia formation, and a theory of ROS-induced sclerotia development was therefore widely recognized (21).

In this theory, vegetative hyphae (an undifferentiated state) need to be stimulated by endogenous ROS stress to initiate hyphal differentiation. Similarly, protective mechanisms such as melanin synthesis and catalase activities would be upregulated to reduce the damage of ROS burst. The sclerotia development ends up with a programmed cell death and autolysis to form the ${\text{O}}_{2}^{-}$-impermeable and melanized rind, while the inner medulla maintains a resting status, waiting for germination in suitable conditions (21). With the advent of high-throughput sequencing in profiling fungal transcriptomes, several studies have examined whether the theory of ROS-induced sclerotia development is universal for the microsclerotia of V. dahliae and Nomuraea rileyi. For example, a study on superoxide dismutase 1 (SOD1), catalase, and glutathione metabolism-related genes proved them to be highly expressed in microsclerotia formation of N. rileyi (32). In addition, one catalase (VDAG_03079) of V. dahliae was found to have higher expression in the microsclerotia-forming strain than the non-microsclerotia-forming strain (33). Consistently, another study reported that three catalases (VDAG_03079, VDAG_03661, and VDAG_09115) were upregulated, with VDAG_03079 being the predominant gene (34). Accordingly, these expression analyses supported that ROS burst and detoxification are associated with microsclerotia formation.

Although the theory of ROS-induced microsclerotia development has been supported using different fungal systems, these fungi all belong to the Leotiomycetes and Sordariomycetes class, while M. phaseolina belong to the Dothideomycetes. It remains unclear whether the mechanism of microsclerotia formation remains conserved among Leotiomycetes, Sordariomycetes, and Dothideomycetes, and it remains uncertain whether the theory of ROS-induced sclerotia development can be applied to the microsclerotia of M. phaseolina. As limited genetic studies have been focused on M. phaseolina, this study applied RNA-sequencing (RNA-Seq) to profile gene expression during the microsclerotia formation stages of M. phaseolina. The differential gene expression and gene ontology analysis identified ROS-related activities during the microsclerotia formation. The microsclerotia inhibition and stimulation assays were conducted and confirmed the involvement of ROS, especially for superoxide (${\text{O}}_{2}^{-}$), as the main stimulus to initiate hyphal differentiation to microsclerotia formation of M. phaseolina.

## RESULTS

### RNA-Seq analyses for the four developmental stages of microsclerotia.

**FIG 1:**
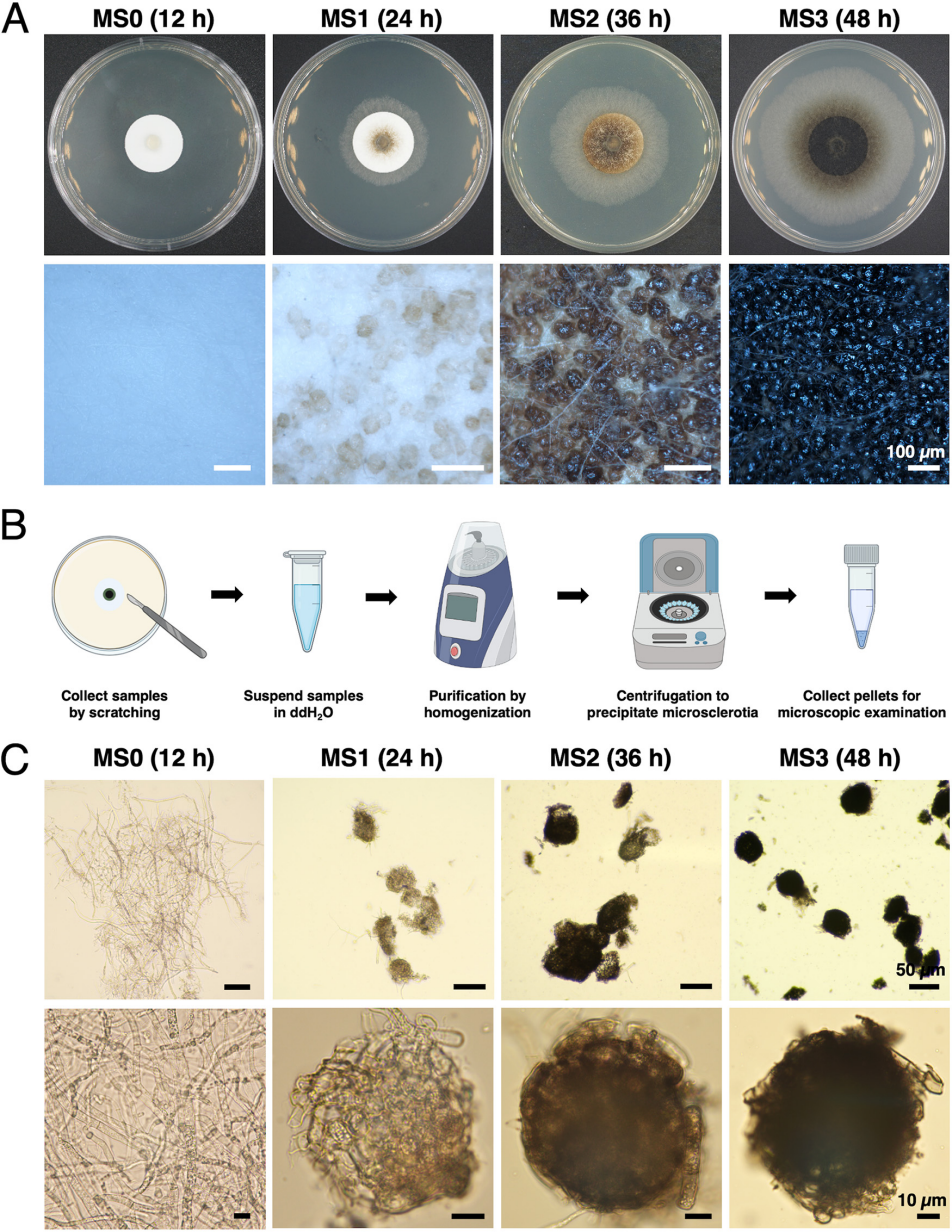
Microsclerotia formation and purification for M. phaseolina. (A) Microsclerotia formation of M. phaseolina categorized into four stages based on the appearance and timeline. MS0 represents the hyphae stage, which contains no pigmentation or aggregation. MS1 represents the initial stage of microsclerotia formation, during which hyphae aggregated into hyaline and spheric structures. MS2 represents the middle stage of microsclerotia formation, during which the spheric structures begin to condense and exhibit brown pigmentation. MS3 represents the mature stage of microsclerotia formation, during which the spheric structures become completely condensed and melanized with reflective mucilage in appearance. (B) Schematic workflow for collecting and purifying microsclerotia. Figures created with BioRender.com. (C) Purified microsclerotia under microscopy. Minimizing mycelia surrounding microsclerotia or attaching on the surface of microsclerotia would provide better representation for the expression patterns in RNA-Seq results. ddH_2_O, double-distilled water.

The microsclerotia formation of M. phaseolina was categorized into four stages: the MS0 stage represented by hyaline and filamentous hyphae showing no wrapping, no swelling, or any specialized structures; the MS1 stage represented by wrapped and aggregated mycelia as the initial form of microsclerotia, but the initial microsclerotia remains white to light brown in color; the MS2 stage represented by light to dark brown microsclerotia; and the MS3 stage represented by condensed and dehydrated microsclerotia with a black appearance and reflective mucilage on the surface (Fig. 1A). Fungal tissues from the four stages of microsclerotia formation were collected at 12, 24, 36, and 48 h postinoculation (hpi). In order to obtain pure microsclerotia of each stage and to minimize the contamination of hyphae surrounding microsclerotia or attached on the surface of microsclerotia, a cleanup workflow was developed to ensure the purity of microsclerotia in each stage (Fig. 1B). Three biological replicates of the hyphae from the MS0 stage, as well as the purified microsclerotia from the MS1, MS2, and MS3 stages, were collected for RNA preparation (Fig. 1C).

**FIG 2:**
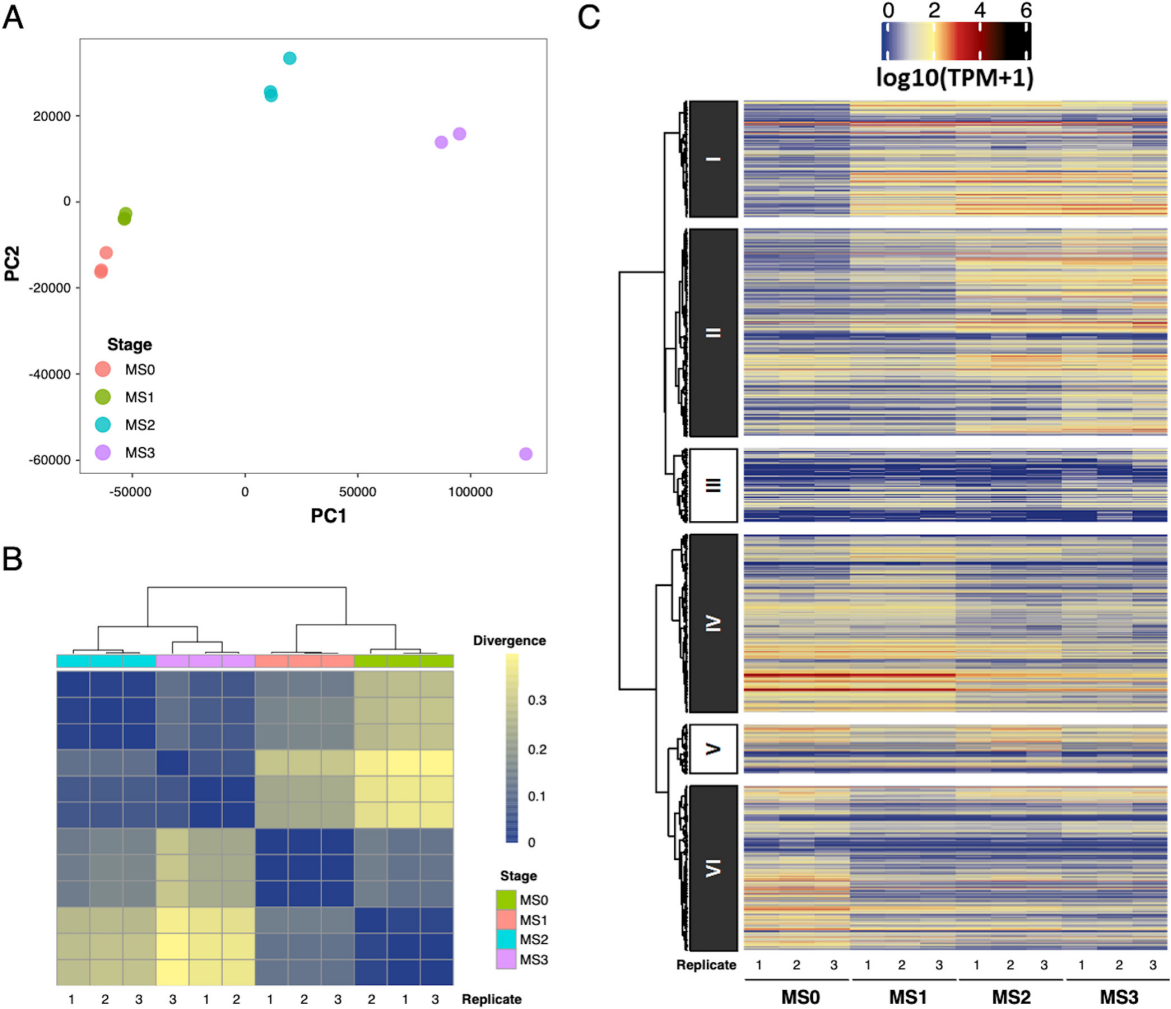
Principal-component analysis (PCA), Jensen-Shannon divergence heat map, and hierarchical clustering for M. phaseolina transcripts. (A) PCA of 12 RNA-Seq samples showed clear separation among four development stages, which indicated good quality of these samples for comparing differential gene expressions between stages. The eigenvalues of PC1 and PC2 were 89.95% and 5.78%, respectively. (B) Jensen-Shannon divergence heat map of RNA-Seq samples based on transcripts per millions (TPM). Lower divergence values represent higher similarity. The transcripts expressed in the hyphae stage and the MS1 stage were closer to each other, while the transcripts expression in the MS2 and MS3 stages were closer to each other. (C) Expression clustering for 12,241 transcripts resulted in six clusters with different expression patterns. Transcripts in clusters I, II, IV, and VI contained reactive oxygen species (ROS)-related functions. While transcripts in clusters I and II were generally upregulated in the later development stages, transcripts in clusters IV and VI were generally downregulated in to the later development stages.

The RNA-Seq analyses resulted in a clear sample separation in the principal-component analysis (PCA) (Fig. 2A; Table S1). The Jensen-Shannon divergence analysis suggested that the samples of MS0 and MS1 had a closer expression pattern, while the samples of MS2 and MS3 had a closer expression pattern (Fig. 2B). These results indicated that the sample purity and experimental setup were qualified for downstream studying the differential gene expressions between the stages of microsclerotia formation. Among 13,443 transcripts in the reference transcriptome, there were 12,241 transcripts detected in the RNA-Seq samples. The hierarchical clustering for these 12,241 transcripts identified six expression patterns, including 1,800, 3,220, 1,140, 2,761, 764, and 2,556 transcripts for clusters I to VI, respectively (Table S2 to S7). Among these six clusters, ROS-related transcripts could be found in clusters I, II, IV, and VI (Fig. 2C).

Cluster I contained transcripts that in general became upregulated in the MS1, MS2, and MS3 stages compared to the MS0 stage. ROS-related transcripts such as Al1_v1_10208 (glutathione *S*-transferase) and Al1_v1_11460 (glutaredoxin) were grouped in this cluster. The cluster II contained transcripts that in general became upregulated in the MS2 and MS3 stage compared to the MS0 stage, and the ROS-related transcripts such as Al1_v1_02200 (catalase) and Al1_v1_12892 (catalase) were grouped in this cluster. On the other hand, cluster IV contained transcripts that in general became downregulated in the MS2 and MS3 stage compared to the MS0 stage, and ROS-related transcripts such as Al1_v1_01834 (superoxide dismutase 1) and Al1_v1_08083 (NADH-ubiquinone oxidoreductase) were grouped in this cluster. Lastly, the cluster VI contained transcripts that in general became downregulated in the MS1, MS2, and MS3 stages compared to the MS0 stage. ROS-related transcripts such as Al1_v1_11257 (lignin peroxidase) and Al1_v1_00390 (lignin peroxidase) were grouped in this cluster.

**FIG 3:**
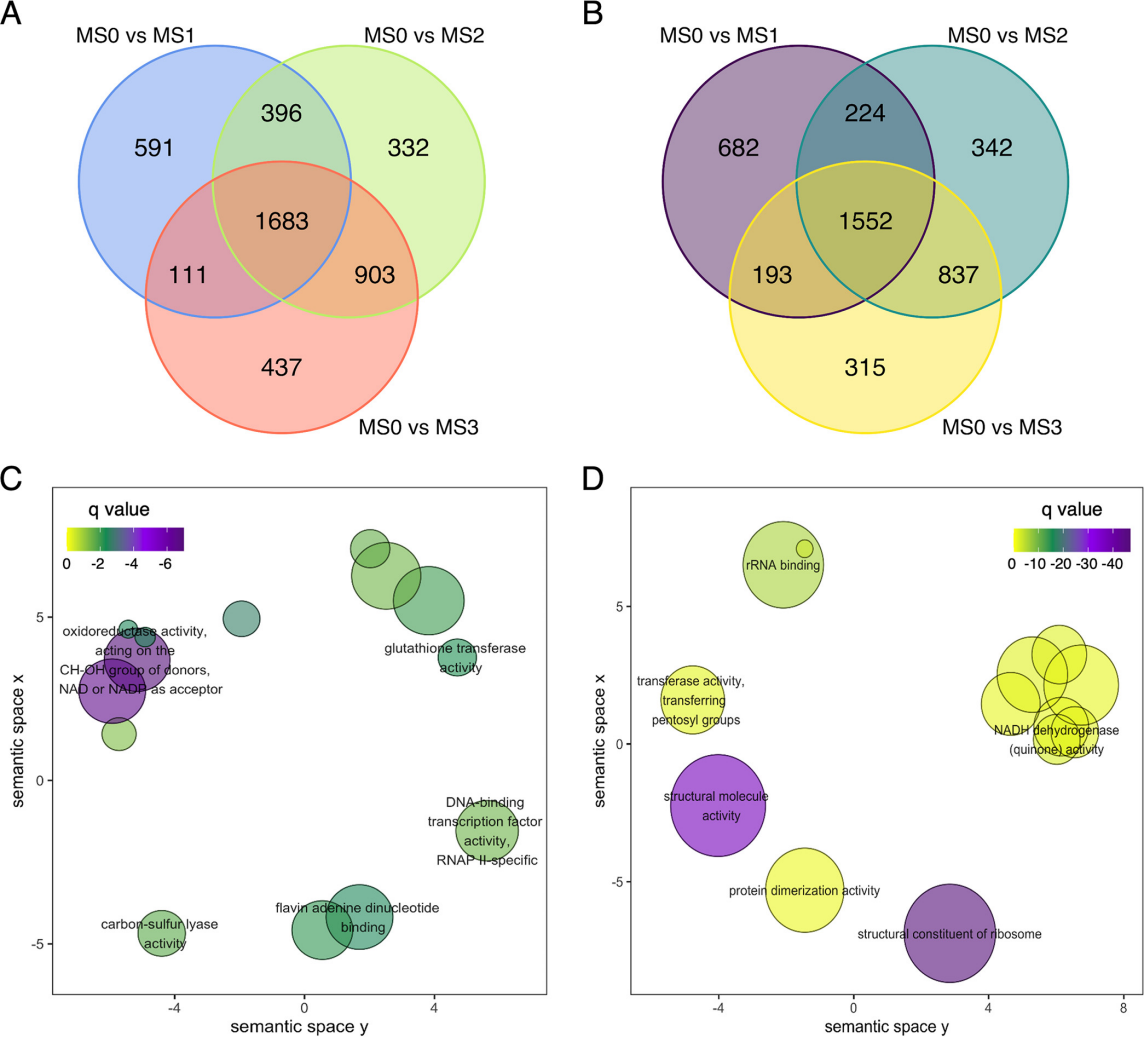
Venn diagram and gene ontology (GO) enrichment of the differential expressed transcripts between the microsclerotia formation stages. (A) Significant upregulated transcripts in the MS1, MS2, and MS3 stages versus the MS0 hyphae stage, respectively. There were 1,683 consensus transcripts found in the three comparisons. (B) Significant downregulated transcripts in the MS1, MS2, and MS3 stages versus the MS0 hyphae stage, respectively. There were 1,552 consensus transcripts found in the three comparisons. (C) GO enrichment for the 1,683 consensus upregulated transcripts identified ROS-related functions such as oxidoreductase and glutathione transferase activities. (D) GO enrichment for the 1,552 consensus downregulated transcripts identified fewer ROS-related functions, such as NADH dehydrogenase activity.

Subsequently, differential expression analyses were performed, and the results showed that there were 2,781, 3,314, and 3,134 transcripts being upregulated in the MS1, MS2, and MS3 over the MS0 stage, respectively. On the other hand, there were 2,651, 2,955, and 2,897 transcripts being downregulated in the MS1, MS2, and MS3 over the MS0 stage, respectively (Table S8 to S10). Venn diagram analysis identified that there were 1,683 transcripts being upregulated in all three comparisons (Fig. 3A), while there were 1,552 transcripts being downregulated in all three comparisons (Fig. 3B). To further understand functional enrichment on these consensus upregulated and downregulated transcripts, gene ontology (GO) analysis was applied, and the results identified several significant biological, molecular, and cellular categories (Table S11 and S12). For the consensus upregulated transcripts, functions such as oxidoreductase activity and glutathione transferase activity were found to be significantly enriched. Other functions, such as the NAD or NADP as acceptor, were also found to be enriched in the upregulated transcripts (Fig. 3C). On the other hand, the NADH dehydrogenase (quinone) activity in the downregulated transcripts was found to be significant, indicating that potential electron-changing functions were active during the microsclerotia formation (Fig. 3D). These results suggested that ROS-related activities and functions were associated with microsclerotia formation of M. phaseolina.

### Antioxidant inhibition assay for microsclerotia formation.

**FIG 4:**
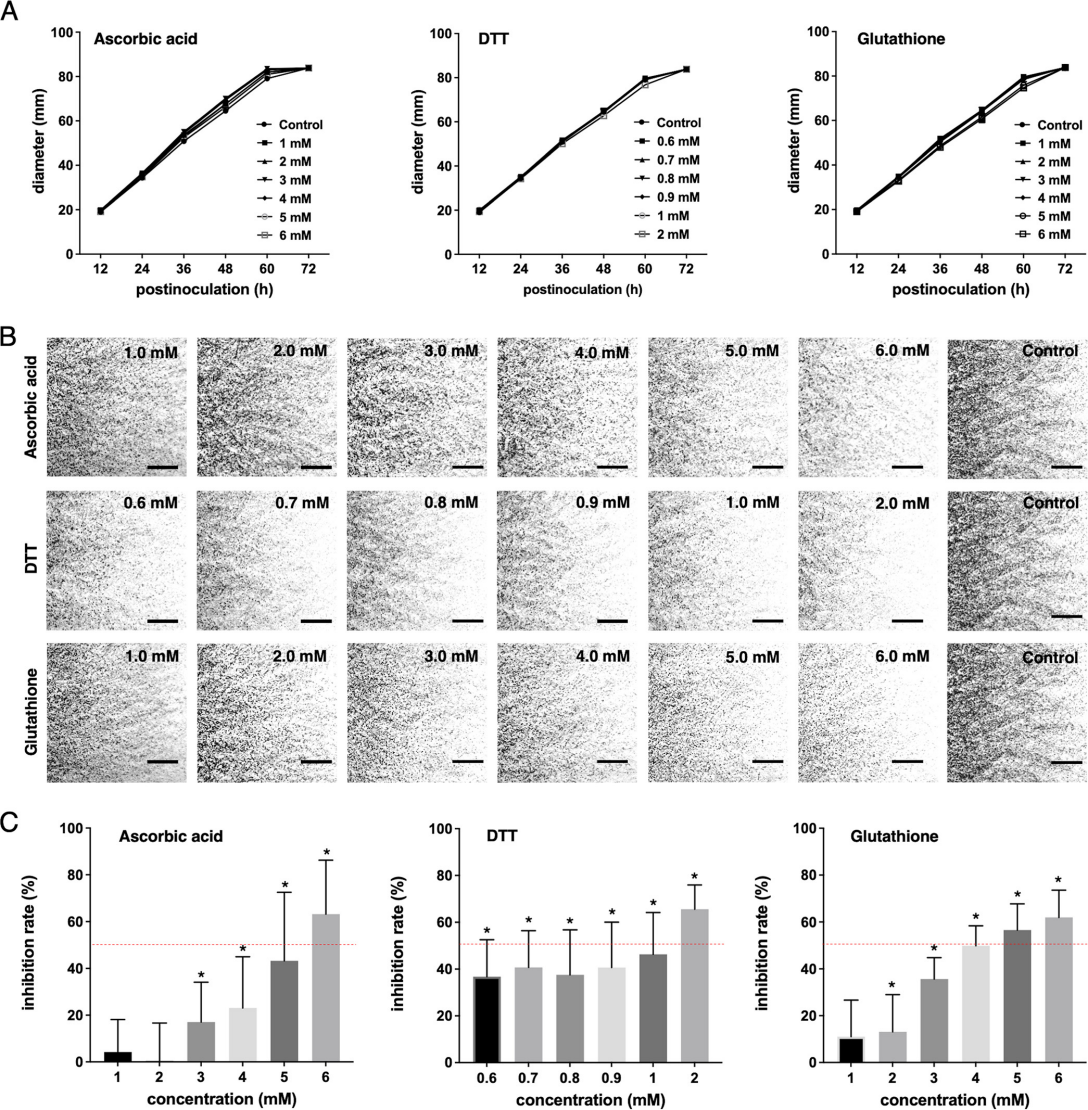
Microsclerotia inhibition assay on the antioxidant-amended PDA plates. (A) Serial concentrations of ascorbic acid, dithiothreitol (DTT), and glutathione were tested, and none of these concentrations showed a negative impact on the mycelia growth rate. (B) Microscopic examination for the microsclerotia formation. (C) Microsclerotia inhibition can be observed when the concentration was higher than 3 mM for ascorbic acid, 0.6 mM for DTT, and 2 mM for glutathione. The asterisks indicate statistical significance at α = 0.05 using *t* test by comparing each concentration to the control. The 50% microsclerotia inhibition (red dashed line) was estimated at 5.5 mM for ascorbic acid, 1 mM for DTT, and 4.5 mM for glutathione, respectively. The experiments contained three repeats, and each repeat contained three biological replicates.

To further validate the importance of ROS in the microsclerotia formation of M. phaseolina, antioxidants including ascorbic acid, DTT, and glutathione were selected to evaluate the effects of antioxidants on the microsclerotia formation. Serial concentrations of these antioxidants were amended into potato dextrose agar (PDA) with no negative impact on the fungal growth regardless of the concentrations (Fig. 4A). However, microsclerotia formation in the PDA plates amended with these antioxidants was reduced (Fig. 4B). Using the ImageJ quantification and simple linear regression, the 50% inhibition concentration was estimated at 5.5 mM for ascorbic acid, 1 mM for DTT, and 4.5 mM for glutathione, respectively (Fig. 4C).

**FIG 5:**
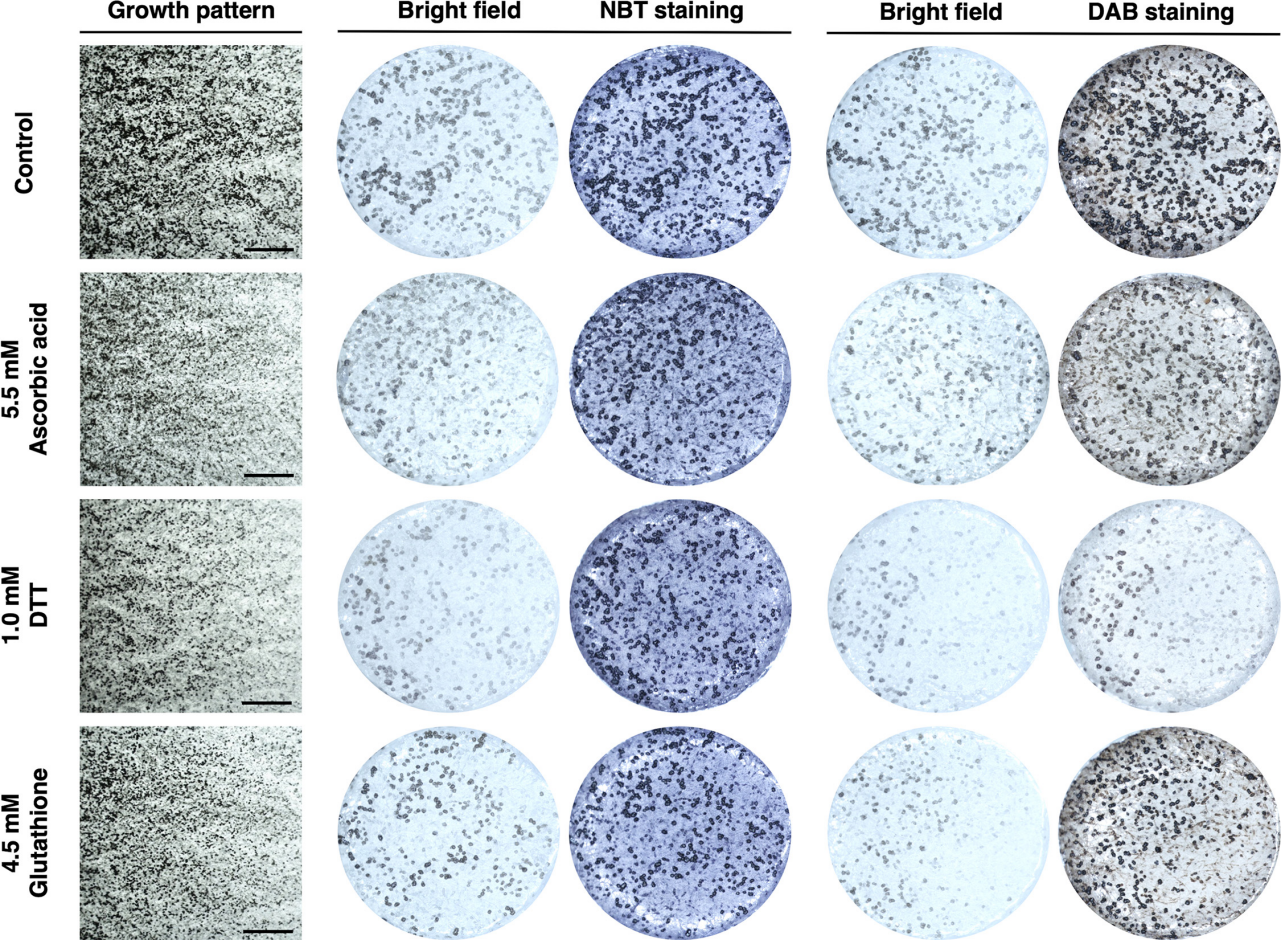
Nitro blue tetrazolium (NBT) staining for ${\text{O}}_{2}^{-}$ and 3,3′-diaminobenzidine (DAB) staining H_2_O_2_ at 50% microsclerotia inhibition on the ascorbic acid-, DTT-, and glutathione-amended potato dextrose agar (PDA) plates. The 5-mm plugs were sampled at 3.5 cm from the center of a 9-cm petri dish for photographing before and after staining. The bars indicate 1 mm. Each staining was repeated two times with three independent biological replicates. At a 50% microsclerotia inhibition rate, the DAB intensity in DTT-amended samples was consistently lower than on ascorbic acid- and glutathione-amended samples, which indicated that lesser amounts of H_2_O_2_ did not impair the microsclerotia formation.

Other than image analyses on the microsclerotia formation, nitro blue tetrazolium (NBT) and 3,3′-diaminobenzidine (DAB) staining were applied to visualize the amount of ${\text{O}}_{2}^{-}$ and H_2_O_2_ in the MS1 stage, respectively (35, 36). At the 50% inhibition rate of microsclerotia formation, the intensity of NBT and DAB were consistently less than the controls, which confirmed that these three antioxidants indeed exhibited ROS scavenging capability. Surprisingly, the DAB staining for H_2_O_2_ in the DTT-amended samples was much lighter than the staining for ascorbic acid- and glutathione-amended samples. However, the NBT staining for ${\text{O}}_{2}^{-}$ in samples of ascorbic acid-, DTT-, or glutathione-amended samples showed no difference (Fig. 5). Because DTT treatment resulted in less H_2_O_2_ (lower intensity of DAB staining) but not a lesser amount of microsclerotia, the importance of H_2_O_2_ in determining the amount of microsclerotia may be minor. In other words, as long as the ${\text{O}}_{2}^{-}$ concentration remained identical, the microsclerotia inhibition rate could be identical at 50% for three antioxidants (Fig. 5).

### ${\text{O}}_{2}^{-}$ and H_2_O_2_ stimulation assay for microsclerotia formation.

**FIG 6:**
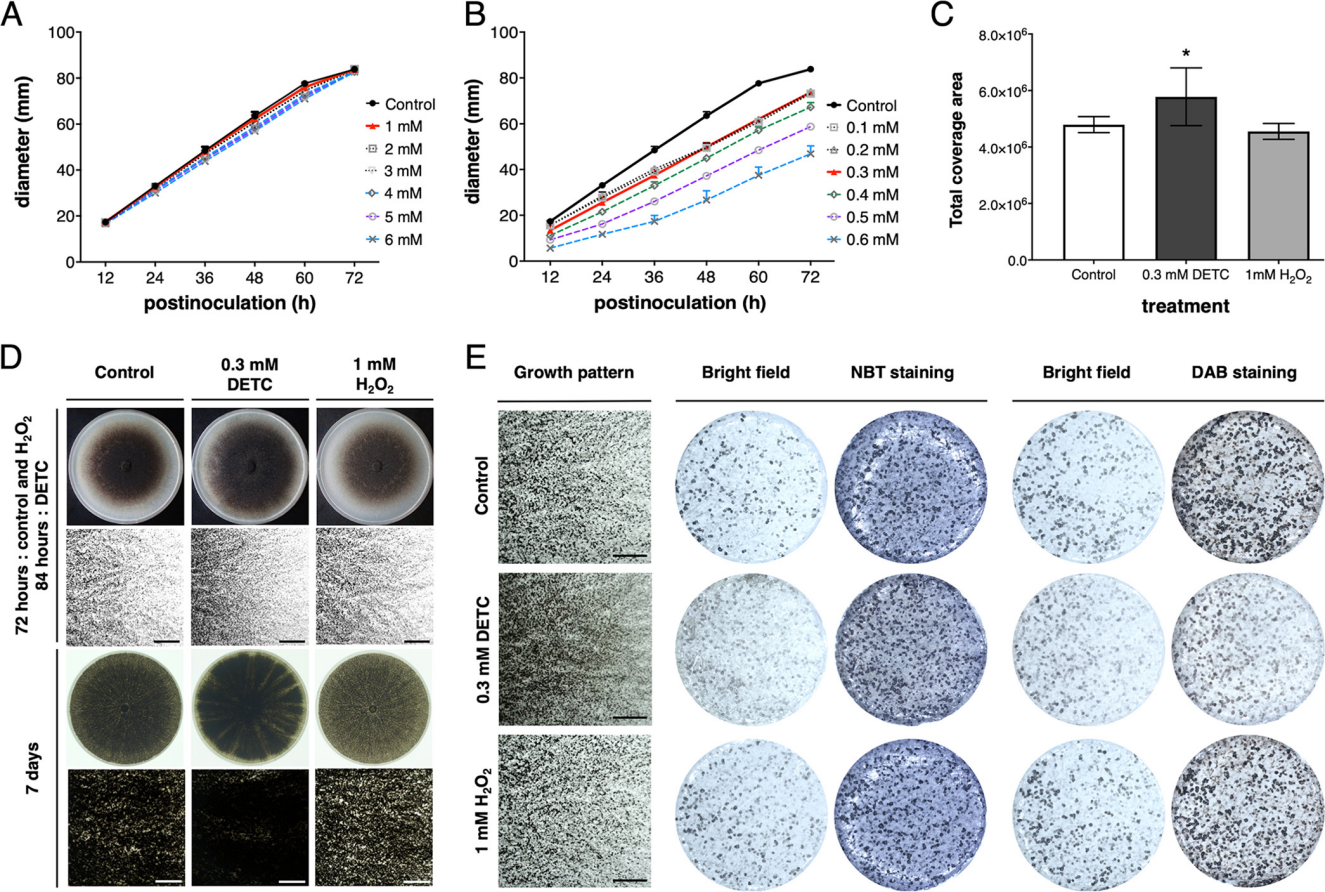
Microsclerotia stimulation assay supported that ${\text{O}}_{2}^{-}$ stimulates microsclerotia formation in M. phaseolina. (A) Mycelial growth rate at different concentrations of H_2_O_2_-amended PDA plates. (B) Mycelial growth rate at different concentrations of diethyldithiocarbamate trihydrate (DETC)-amended PDA plates. The best concentration was defined as the highest concentration not affecting mycelial growth, as indicated by the red line, which was 0.3 mM for DETC and 1 mM for H_2_O_2_. (C) Microsclerotia stimulation rate of 0.3 mM DETC and 1 mM H_2_O_2_. An increase of microsclerotia formation of approximately 10% was estimated in 0.3 mM DETC-amended PDA plates. (D) Microsclerotia formation in a plate and in the area 3.5 cm from the center of a 9-cm petri dish. The DETC treatment stimulated high microsclerotia formation. Three biological replicates were included in each experimental repeat, and the experiment was repeated three times. The bars indicate 1 mm. (E) 5-mm plugs were sampled at the 3.5 cm from the center of a 9-cm petri dish for photographing before and after staining. The bars indicate 1 mm. Each staining was repeated three times, and each included three independent biological replicates. Because DETC blocks the process of ${\text{O}}_{2}^{-}$ into H_2_O_2_ without reducing microsclerotia formation, the results support that ${\text{O}}_{2}^{-}$ is the main ROS stimulus for microsclerotia formation in M. phaseolina.

In order to further confirm the contribution of ${\text{O}}_{2}^{-}$ and H_2_O_2_ in determining the amount of microsclerotia formation, the Zn/Cu superoxide dismutase SOD1 inhibitor sodium diethyldithiocarbamate trihydrate (DETC) and H_2_O_2_ were amended to provide the ROS stress, specifically for increasing ${\text{O}}_{2}^{-}$ and H_2_O_2_, respectively. Different concentrations of DETC and H_2_O_2_ were evaluated in PDA to determine the highest concentration that can be amended without harming fungal growth. However, both DETC and H_2_O_2_ caused growth rate reduction in a concentration-dependent manner. Among the tested conditions, H_2_O_2_ at 1 mM showed no significant difference from the control (Fig. 6A). On the other hand, the concentrations of 0.1, 0.2, and 0.3 mM caused the same level of growth reduction but no significant difference among these three concentrations (Fig. 6B). As lower concentrations still exhibited negative impacts on mycelial growth, DETC at 0.3 mM was selected to observe its effects on the microsclerotia formation. The growth rate for M. phaseolina was measured at 1.29 ± 0.02, 1.27 ± 0.04, and 1.03 ± 0.02 mm/h on the control PDA plates, on the 1 mM H_2_${\text{O}}_{2}^{-}$amended plates, and on the 0.3 mM DETC-amended plates, respectively. In order to compensate the reduction of growth rate, the experiments were recorded at 72 hpi for control and 1 mM H_2_O_2_-amended plates and 84 hpi for 0.3 mM DETC-amended plates. At these time points, the mycelia were about covered the whole 9-cm petri dish. In addition, these plates were prolongedly cultured to double confirm the final amount of microsclerotia after 7 days.

The 0.3 mM DETC-amended plates increased microsclerotia formation, while the 1 mM H_2_O_2_-amended plates showed no significant difference to the control (Fig. 6C). When the growth of M. phaseolina covered the DETC-amended PDA plate, microsclerotia formation showed high density and a wider melanized zone compared to the control, and the increased amount of microsclerotia was further confirmed after 7 days. On the other hand, the microsclerotia formation of M. phaseolina on the H_2_O_2_-amended PDA plate showed no clear difference to the control at 72 hpi, but a clear reduction of microsclerotia could be observed after 7 days (Fig. 6D). Furthermore, the DAB staining confirmed that the addition of SOD1 inhibitor DETC was functional to block the metabolic conversion from ${\text{O}}_{2}^{-}$ to H_2_O_2_, and the intensity of H_2_O_2_ was much less compared to the H_2_O_2_-amended samples and controls (Fig. 6E). However, the microsclerotia formation was increased in the DETC-amended plates. Consistent with the ROS antioxidant inhibition assay, these results supported the role of ${\text{O}}_{2}^{-}$, but not H_2_O_2_, in initiating the differentiation from hyphae to microsclerotia formation.

### Expression patterns of ROS-related genes in the four microsclerotia formation stages.

**FIG 7:**
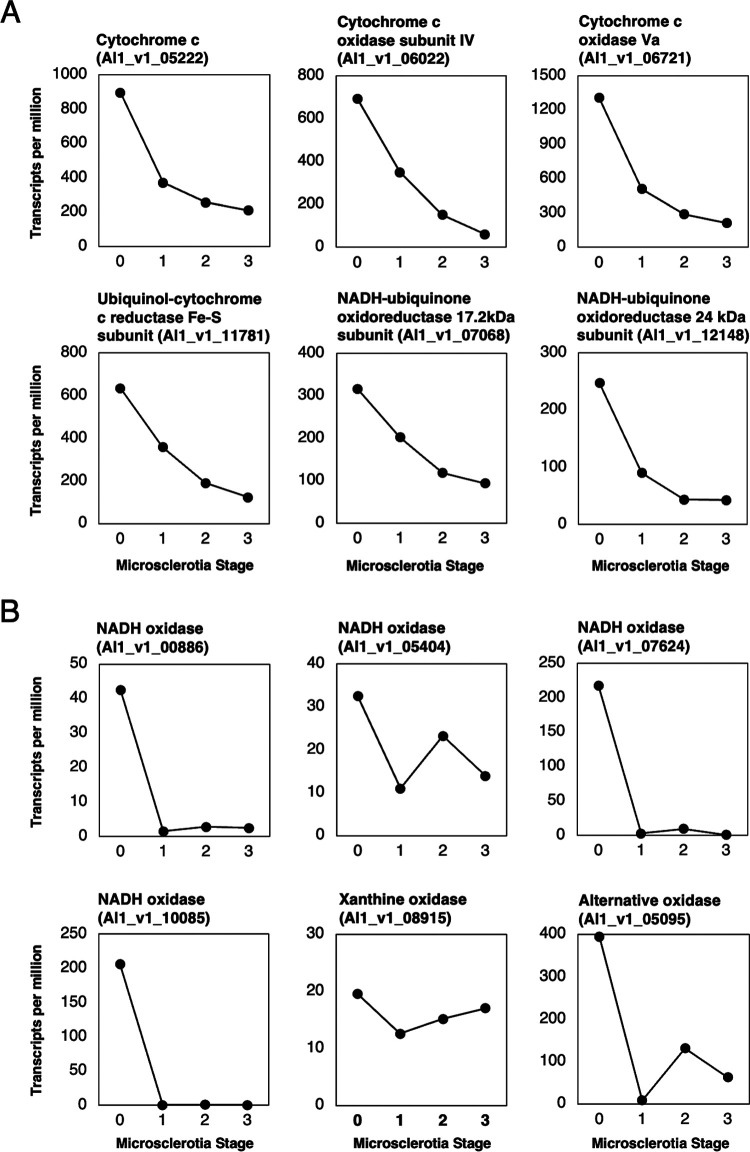
Expression patterns of transcripts that may generate ${\text{O}}_{2}^{-}$ during microsclerotia formation. (A) Cytochrome *c* and NADH-ubiquinone oxidoreductase are known to involve in the electron transport chain to generate ${\text{O}}_{2}^{-}$. Six transcripts annotated as cytochrome *c* or NADH-ubiquinone oxidoreductase were found to have higher expression in the MS0 stage. (B) NADPH oxidase, xanthine oxidase, and alternative oxidase are also known to involve in the generation of ${\text{O}}_{2}^{-}$. Six transcripts annotated as NADPH oxidase, xanthine oxidase, or alternative oxidase exhibited higher expression in the MS0 stage. These results indicated that ${\text{O}}_{2}^{-}$ generation needs to be active before the initial stage of microsclerotia formation.

With the knowledge that ${\text{O}}_{2}^{-}$ initiates hyphal differentiation to microsclerotia of M. phaseolina, transcripts with ROS-related function in the ROS detoxification pathways were further evaluated. It has been shown that the ${\text{O}}_{2}^{-}$ stress could be produced by the enzymes in the mitochondrial electron transport chain such as the cytochrome *c* oxidases and NADH-ubiquinone-related oxidoreductases (37, 38). There were four cytochrome *c*-related transcripts, including cytochrome *c* (Al1_v1_05222), cytochrome *c* oxidase subunit IV (Al1_v1_06022), cytochrome *c* oxidase Va (Al1_v1_06721), and ubiquinol-cytochrome *c* reductase Fe-S subunit (Al1_v1_11781), as well as two NADH-ubiquinone oxidoreductases (Al1_v1_07068 and Al1_v1_12148) that exhibited the highest expression at the MS0 stage (Fig. 7A; Table S13). In addition, the ${\text{O}}_{2}^{-}$ stress may be produced by membrane-associated enzymes such as NADPH oxidases, xanthine oxidases, and alternative oxidases (39). There were four NADPH oxidases (Al1_v1_00886, Al1_v1_05404, Al1_v1_07624, and Al1_v1_10085), xanthine oxidase (Al1_v1_08915), and alternative oxidase (Al1_v1_05095), which also showed the highest expression at the hyphal stage (Fig. 7B). The observation supported the results that ${\text{O}}_{2}^{-}$ stress accumulated at the MS0 to MS1 stage to stimulate microsclerotia formation and determining the amount of microsclerotia being formed. Upon the completion of hyphae aggregation at the MS1 stage, detoxification of ${\text{O}}_{2}^{-}$ and H_2_O_2_ stress would be needed.

**FIG 8:**
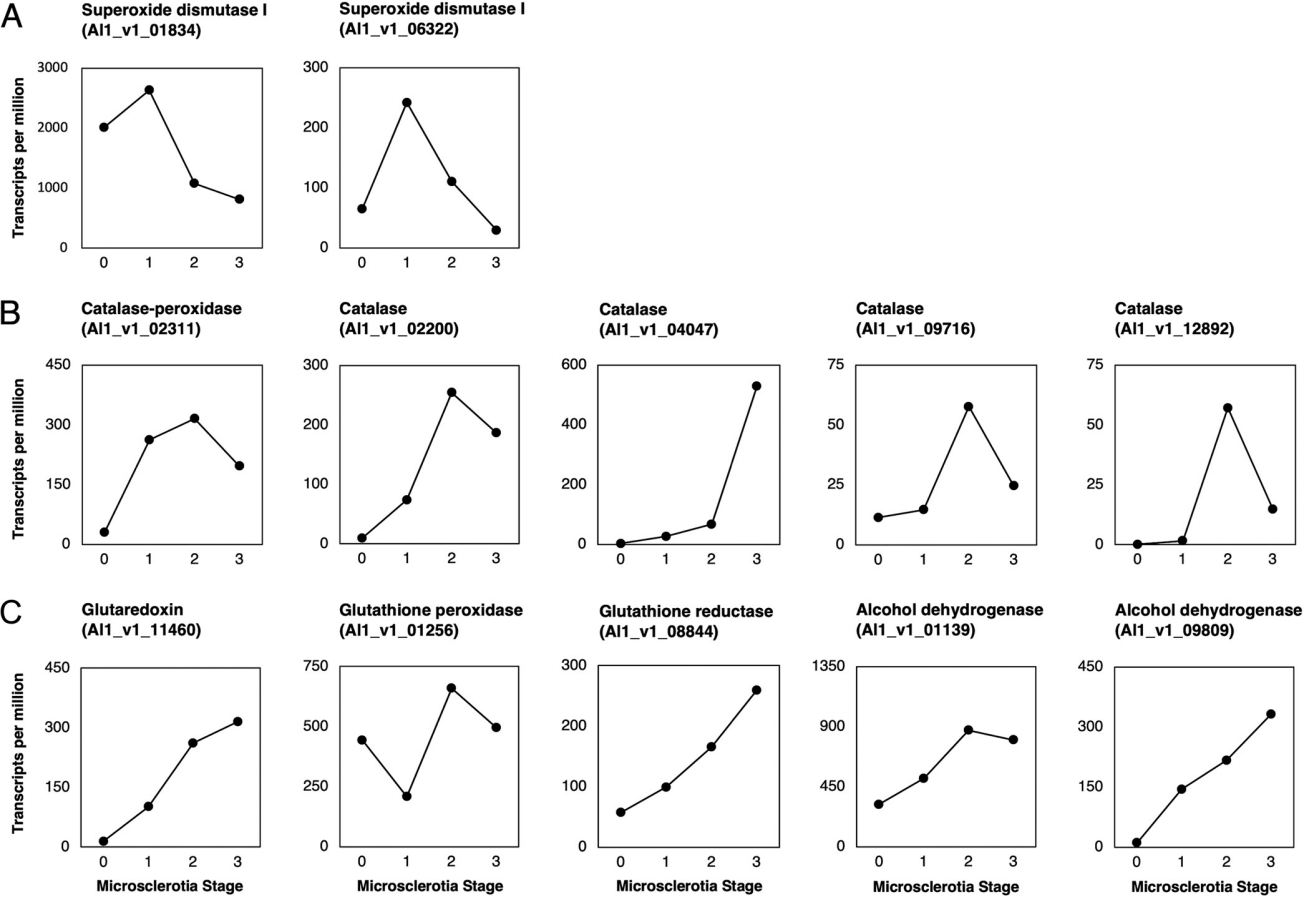
Expression patterns of transcripts that may detoxify ${\text{O}}_{2}^{-}$ or H_2_O_2_ during microsclerotia formation. (A) Two transcripts annotated as superoxide dismutase 1 exhibited highest expression at the MS1 stage, which indicated that the ${\text{O}}_{2}^{-}$ stress was higher at the MS0 to MS1 stage. (B) Five transcripts annotated as catalases showed higher expression toward the MS2 or MS3 stage, which indicated that the H_2_O_2_ stress was higher after the MS1 stage. (C) Glutaredoxin in the glutaredoxin pathway and four transcripts in the glutathione pathway were also found to have higher expression toward the MS2 or MS3 stage, which supported the H_2_O_2_ stress being higher at later stages.

**FIG 9:**
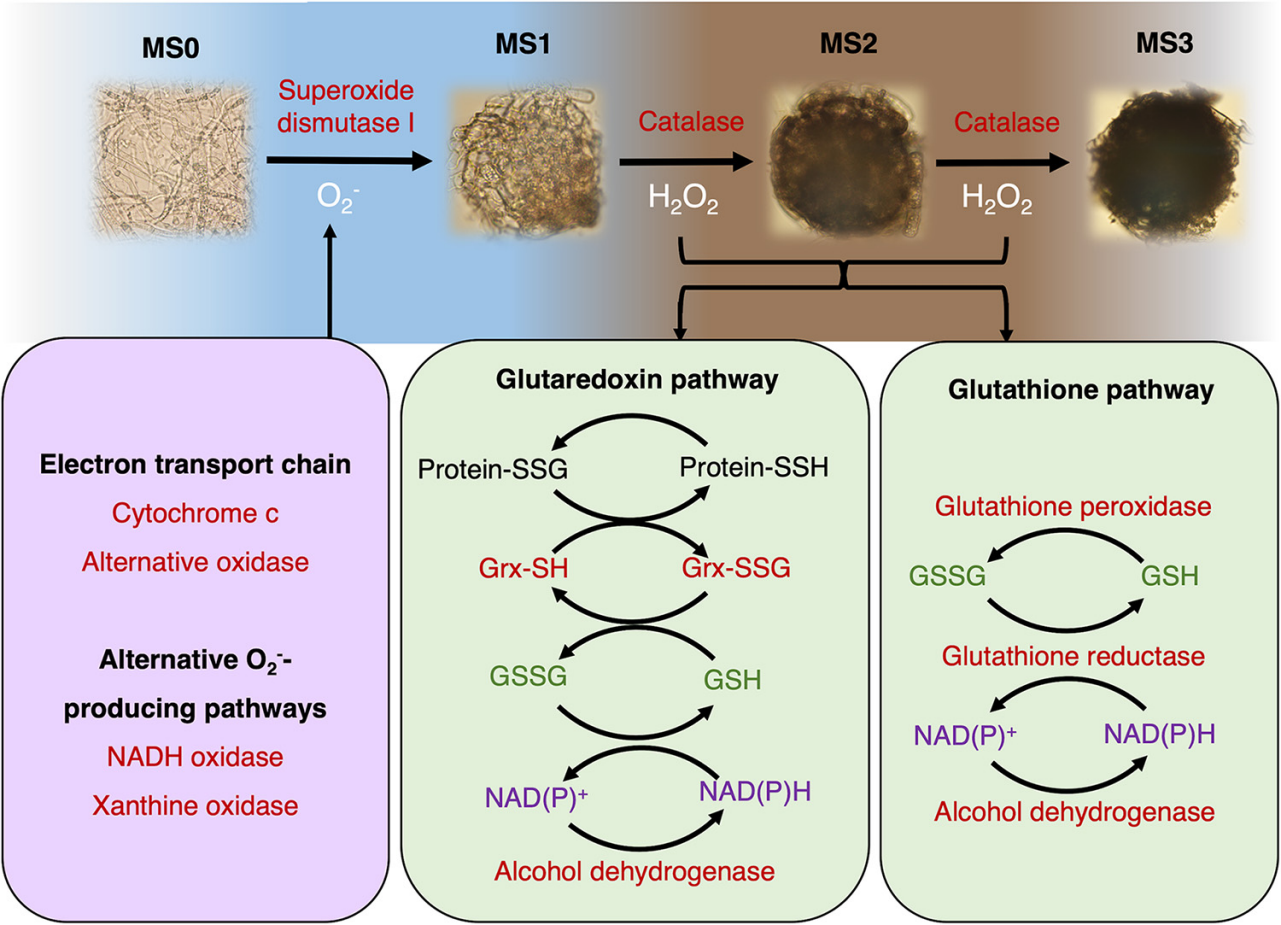
The schematic model for ROS metabolism during microsclerotia formation of M. phaseolina. ${\text{O}}_{2}^{-}$ generated from electron transport chain and alternative pathways serves as the main ROS stimulus to initiate hyphal differentiation into the initial stage of microsclerotia formation (MS1). ${\text{O}}_{2}^{-}$ detoxification is mediated by superoxide dismutase I, which converts ${\text{O}}_{2}^{-}$ to H_2_O_2_. The accumulation of H_2_O_2_ in the middle stage of microsclerotia formation (MS2) may facilitate development into the mature stage of microsclerotia (MS3). Downstream metabolisms, including melanin synthesis and H_2_O_2_ detoxification through catalases, glutaredoxin pathway, and glutathione pathway, will be upregulated to prevent ROS damage to the resting fungal tissues.

To detoxify ${\text{O}}_{2}^{-}$ stress, the Zn/Cu SOD1 has been shown to process ${\text{O}}_{2}^{-}$ into H_2_O_2_ (40, 41). There were two Zn/Cu SOD1 genes (Al1_v1_01834 and Al1_v1_06322) in the M. phaseolina genome, and both genes reached the highest expression at the MS1 stage and gradually dropped down at the MS2 and MS3 stages. The phenomenon indicated that the ${\text{O}}_{2}^{-}$ stress was high at the MS0 to MS1 stages, and therefore, two Zn/Cu SOD1 genes needed to be upregulated to convert ${\text{O}}_{2}^{-}$ into H_2_O_2_ (Fig. 8A). On the other hand, it has been shown that catalases, the glutaredoxin pathway, and the glutathione pathway could reduce H_2_O_2_ stress (42). There were one catalase-peroxidase (Al1_v1_02311) and four catalases (Al1_v1_02200, Al1_v1_04047, Al1_v1_09716, and Al1_v1_12892), and these five genes reached the highest expression at the MS2 or MS3 stage, which suggested that H_2_O_2_ stress was higher after the MS1 stage (Fig. 8B). In addition, five enzymes, including glutaredoxin (Al1_v1_11460), glutathione peroxidase (Al1_v1_01256), glutathione reductase (Al1_v1_08844), and two alcohol dehydrogenases (Al1_v1_01139 and Al1_v1_09809), were also upregulated at the MS2 or MS3 stage (Fig. 8C). These results supported the possibility that the H_2_O_2_ stress comes after the MS1 stage, and therefore ${\text{O}}_{2}^{-}$ serves as the initial ROS stimulus to initiate hyphal differentiation into microsclerotia for M. phaseolina. After the hyphal aggregation and the formation of hyaline microsclerotia, the ${\text{O}}_{2}^{-}$ detoxification by SOD1 and H_2_O_2_ detoxification by catalases and enzymes in the glutaredoxin pathway and glutathione pathway became active to relieve downstream ROS stresses (Fig. 9).

## DISCUSSION

Sclerotia or microsclerotia formation is a survival strategy for plant-pathogenic fungi such as S. sclerotiorum, S. minor, V. dahliae, and M. phaseolina. Advanced studies on the sclerotia and microsclerotia biology are desired in order to better manage these soilborne diseases by reducing the pathogen inoculum surviving in debris and soils (5, 43, 44). Although the theory of ROS-induced sclerotia development has been supported by studying Leotiomycetes and Sordariomycetes using transcriptomic analysis and ROS inhibition assay, the theory in microsclerotia of V. dahliae and N. rileyi has been evaluated only using transcriptomic analyses; ROS inhibition and stimulation assay in the microsclerotia formation have been lacking. Moreover, M. phaseolina belongs to the Dothideomycetes, which is different from V. dahliae and N. rileyi, that both belong to the Sordariomycetes. Therefore, it remained unclear whether ROS stimulation remains conserved for the microsclerotia formation in M. phaseolina. This study applied RNA-Seq to profile four stages of microsclerotia formation in M. phaseolina, and the expression analyses identified that ROS-related transcripts were differentially expression during the microsclerotia formation stages. Using the ROS inhibition assay and stimulation assay, this study further demonstrated that ${\text{O}}_{2}^{-}$ is the main ROS stimulus to initiate hyphal differentiation for microsclerotia formation of M. phaseolina.

### Studies supporting that ROS generation upstream of ${\text{O}}_{2}^{-}$ and H_2_O_2_ is involved in sclerotia and microsclerotia formation.

There are several pieces of evidence to support that the generation of ${\text{O}}_{2}^{-}$ and H_2_O_2_ are important for sclerotia and microsclerotia formation. For example, NADPH oxidase (Nox) homologs are conserved in eukaryotic cells, and the Nox family has been widely recognized for generating ${\text{O}}_{2}^{-}$. Two studies reported that mutants of S. sclerotiorum NADPH oxidases (SsNox1 and SsNox2) resulted in less sclerotia formation (45, 46). A similar phenomenon was observed for Botrytis cinerea, and the deletion mutants of either BcNoxA or BcNoxB exhibited no sclerotia (47, 48). The N. rileyi NrNOX-silenced mutants showed less microsclerotia formation and decreased H_2_O_2_ accumulation (49). For the V. dahliae NoxA mutants, reduction of microsclerotia formation was also observed (50). However, the product of Nox may differ depending on the homologous lineage, and it has been suggested that the main product of Nox1, Nox2, Nox3, and Nox5 is ${\text{O}}_{2}^{-}$, while the main product of Nox4 is H_2_O_2_ (39). Moreover, the function of Nox may vary depending on the cellular location. For example, other research studying *B. cinerea* suggested that BcNoxA/B on the endoplasmic reticulum produces H_2_O_2_, while BcNoxA and BcNoxB on the plasma membrane generate ${\text{O}}_{2}^{-}$ and H_2_O_2_, respectively (51). Moreover, NBT staining in BcNoxA/B double mutant resulted in similar NBT intensity, which indicated that additional pathways may have functional redundancy to BcNoxA/B in generating ${\text{O}}_{2}^{-}$ (45). Because the conversion of ${\text{O}}_{2}^{-}$ to H_2_O_2_ in the ROS detoxification pathway is dynamic, studies on the upstream genes such as Nox will interfere with both ${\text{O}}_{2}^{-}$ and H_2_O_2_ simultaneously. Therefore, a better experimental strategy may be evaluating the detoxification of ${\text{O}}_{2}^{-}$ and H_2_O_2_ by characterizing SOD1 and catalases.

### Studies supporting ${\text{O}}_{2}^{-}$ as the main stimulus for sclerotia and microsclerotia formation.

In studying the sclerotia formation of other fungi such as S. minor and S. sclerotiorum, one study showed that sclerotia formation was reduced by adding SOD mimetics, which convert ${\text{O}}_{2}^{-}$ into H_2_O_2_. In other words, sclerotia formation was reduced if ${\text{O}}_{2}^{-}$ was decreased (29). In measuring the ${\text{O}}_{2}^{-}$ concentration and the xanthine oxidase-derived ${\text{O}}_{2}^{-}$, it was found that ${\text{O}}_{2}^{-}$ concentration was consistently higher in sclerotia-forming isolates than non-sclerotia-forming isolates (28, 30). Consistently, a study showed that S. minor growth on the H_2_O_2_-amended PDA did not result in more sclerotia (52).

One study observed that the mutants of S. sclerotiorum SOD1 (SsSOD1, SS1G_00699) showed no impairment on the sclerotia formation (53), which indicated that the elimination of H_2_O_2_ had no negative impact on sclerotia formation. In addition, two studies silencing catalases of S. sclerotiorum, which conceptually increased H_2_O_2_ accumulation, turned out to reduce sclerotia formation. In silencing the SsCat1 (SS1G_02784), the mutants showed slower and smaller sclerotia formation (54), and in silencing the SsCat2 (SS1G_00547), a reduced number of sclerotia was observed, although the size and weight of the sclerotia appeared to be normal (55). A similar effect was found in Aspergillus flavus, for which the increase of SOD expression and decrease of catalase expression in the *veA* mutant, or applying SOD mimetic TIRON and catalase inhibitor aminotriazole to the wild-type strain, all resulted in the accumulation of H_2_O_2_ with no sclerotia formation (56).

Other than increasing H_2_O_2_ accumulation by silencing catalases, in silencing other H_2_O_2_ detoxification mediators, including the S. sclerotiorum thioredoxin1 SsTrx1 (SS1G_08534) and thioredoxin reductase SsTrr1 (SS1G_05899), both mutants exhibited higher sensitivity and less capability to handle exogenous H_2_O_2_. The results also showed a consistent reduction of the sclerotia number in the SsTrx1-silenced and the SsTrr1-silenced mutants, which indicated that the accumulation of H_2_O_2_ may not promote sclerotia formation (57, 58). Accordingly, these studies suggested the possibility of ${\text{O}}_{2}^{-}$ but not H_2_O_2_ in stimulating and determining the number of sclerotia and microsclerotia formation.

### Studies supporting H_2_O_2_ as the main stimulus for sclerotia and microsclerotia formation.

However, there are also several articles that suggested H_2_O_2_ was important for sclerotia formation, including studies on A. flavus, R. solani, S. sclerotiorum, S. minor, A. rolfsii, and N. rileyi (29, 31, 45). Moreover, in contrast to the knockout study of SsSOD1 (53), a study showed a reduced sclerotia formation when the same SsSOD1 was silenced and when S. sclerotiorum was grown on the DETC-amended PDA plates (59). These results suggested that blocking the generation of H_2_O_2_ resulted in no sclerotia formation, and that the application of exogenous H_2_O_2_ stimulated more sclerotia formation for S. sclerotiorum has also been documented (31). Specifically for microsclerotia formation, a recent study on the V. dahliae SOD1 (VdSOD1) revealed its importance as a secretory protein in scavenging both exogenous and endogenous ${\text{O}}_{2}^{-}$, but the phenotype of microsclerotia formation was not described in the VdSOD1 mutants (60).

On the other hand, a study on knocking out A. flavus catalase CTA1 resulted in an increased number of sclerotia, which suggested the accumulation of H_2_O_2_ promoted sclerotia formation (61). Another study silencing the cinnamyl alcohol dehydrogenase of S. sclerotiorum (SsCAD) caused the downregulation of SsNox1 and SsNox2, and less sclerotia formation was observed. The sclerotia formation was partially recovered when the SsCAD-silenced mutant was grown on the 6 mM H_2_O_2_-amended PDA plates, indicating the importance of H_2_O_2_ in stimulating sclerotia formation. However, this study also reported the recovery was better in the 0.2 M NADPH-amended PDA plates, which suggested that a fully functional ${\text{O}}_{2}^{-}$ to H_2_O_2_ detoxification pathway can rescue sclerotia formation better than supplying H_2_O_2_ alone (62).

### ${\text{O}}_{2}^{-}$ as the main stimulus for initiating hyphal differentiation into microsclerotia of M. phaseolina.

While many studies have provided evidence for the importance of ${\text{O}}_{2}^{-}$ or H_2_O_2_ in the sclerotia or microsclerotia formation in studying different Leotiomycetes and Sordariomycetes, this study integrated not only transcriptomic analyses, but also the ROS inhibition and stimulation assay to support the involvement of ROS-related activities during the microsclerotia formation of M. phaseolina in the Dothideomycetes class. Moreover, by using NBT staining for ${\text{O}}_{2}^{-}$ and DAB staining for H_2_O_2_, this study provided a direct visualization for the importance of ${\text{O}}_{2}^{-}$ in determining the amount of microsclerotia formation. As M. phaseolina growing on the SOD1 inhibitor DETC-amended PDA plates resulted in more microsclerotia formation, but not M. phaseolina growing on the H_2_O_2_-amended PDA plates, the results provided direct supports for ${\text{O}}_{2}^{-}$ as the main ROS stimulus in initiating microsclerotia formation of M. phaseolina. The results also suggested that the ROS stimulation mechanism or ROS signal may remain conserved in the evolution between Leotiomycetes, Sordariomycetes, and Dothideomycetes. Future studies would further resolve the details in ${\text{O}}_{2}^{-}$ and/or H_2_O_2_ reception and additional mechanisms involved in the sclerotia- or microsclerotia-forming capability of plant-pathogenic fungi.

## MATERIALS AND METHODS

### Fungal material and microsclerotia preparation.

M. phaseolina isolate a31 was routinely maintained on PDA in dark at 28°C. The Mycelia blocks from actively growing margins of colonies were inoculated on PDA covered with a 0.45-μm nylon membrane (Pall Corporation, Ann Arbor, MI). Sample collection was completed at four stages of microsclerotia formation. The first stage (MS0) collected the mycelia at 12 h postinoculation (hpi) from potato dextrose broth after filtering mycelia through a double layer gauze with twice washing using sterile water. The second stage (MS1), third stage (MS2), and fourth stage (MS3) collected microsclerotia at 24, 36, and 48 hpi, respectively, by scratching fungal tissue from the nylon membrane on PDA using a scalpel, and the fungal tissues were placed into a 2-mL screw cap prefilled with 1 mL sterile water and 1.0 mm zirconium dioxide beads. In order to remove hyphae and to obtain clean microsclerotia, the samples were gently vortexed using the Bioprep-BM24 instrument (Bioman Scientific Co., Ltd., New Taipei City, Taiwan) at 4.0 m/s speed for 20 s for MS1 samples, 5.0 m/s speed 30 s for MS2 samples, and 2 min without beads for MS3 samples. The samples were centrifuged at 14,000 rpm for 30 s, and the supernatants were discarded. The pellets were washed with 1 mL sterile water again before a centrifugation at 14,000 rpm for 1 min. The samples were assessed under a stereo dissecting microscope, and samples of good integrity and cleanness were immediately frozen in liquid nitrogen and stored in the −80°C freezer.

### RNA-Seq preparation.

The frozen samples were thawed by adding 1 mL of TRIzol (Invitrogen, Thermo Fisher Scientific Inc., Waltham, MA) and homogenized in a mortar with a pestle. The fungal nucleic acids were precipitated using isopropanol after cleaning by chloroform twice. The pellets were washed by 75% ethanol and resuspended in the UltraPure diethyl pyrocarbonate (DEPC)-treated water (Invitrogen). The fungal DNA was removed by the TURBO DNA-*free* kit (Invitrogen), and the fungal RNA was cleaned up and harvested by the RNA cleanup kit (Geneaid, New Taipei City, Taiwan) following the manufacturers’ manuals. The integrity of purified RNA was qualified by the Bioanalyzer 2100 instrument (Agilent Technologies, Inc., Santa Clara, CA), and the library construction was completed using the Illumina MiSeq reagent kit v3 (Illumina, San Diego, CA). The 12 RNA libraries, which includes 3 biological replicates for the four stages of microsclerotia formation, were sequenced using the 150-bp single-end option of Illumina MiSeq (Illumina) in the Center of the Technology Commons (TechComm) in the College of Life Sciences at National Taiwan University.

### Differential expression and gene ontology (GO) analyses.

The Illumina raw data were quality control using the FASTQC and FASTX-Toolkit (63, 64). Reads with Phred scores above 30 were subjected to the gene expression quantification using the Kallisto version 0.46.1 and sleuth pipeline in default (65, 66). The genome of M. phaseolina Al-1 isolate was used as the reference (67). The differential gene expressions were analyzed by comparing the MS1, MS2, and MS3 stages to the MS0 stage, and the significance was determined at *q* value below 0.05. The expression patterns were clustered using the Pearson’s distance and Ward.D2 method and visualized using the heat map function implemented in the R package “ComplexHeatmap” version 2.8.0 (68). The protein models of M. phaseolina Al-1 genome were annotated according to the OMA orthology database to obtain GO terms (69), and the GO enrichment analysis was analyzed using the R package “clusterProfile” version 4.1 (70). Venn diagram analyses were applied to identify the consensus genes with upregulation and downregulation in the MS1, MS2, and MS3 stages versus the MS0 stage, and the GO of consensus genes were subjected to REVIGO to visualize the functional annotation and statistical significance (71).

### ROS inhibition assay.

The ascorbic acid (PanReac AppliChem, ITW Reagents, Darmstadt, Germany), dithiothreitol (DTT) (BioShop Canada Inc., Burlington, Canada), and glutathione (BioShop) were freshly prepared in the 9-cm petri dish PDA plates to minimize oxidation of the antioxidants. A 5-mm PDA block containing the mycelia edge of M. phaseolina was transferred to the center of the PDA plate, and the culture was continued in dark at 28°C until the mycelia covered the whole plate. The microsclerotia formation was examined and photographed under a stereo dissecting microscope at 240- to 300-fold magnitudes (WHITED Company Ltd., Taipei, Taiwan). Three photos were taken from each plate by photograph parameter adjustments, following exposure target 120, exposure time 41.66 ms, and gain 1006% of the RisingView 3.7 digital camera software, and all conditions (ascorbic acid, DTT, glutathione, and control) were repeated three times with three biological replicates/plates in each repeat. The images were input into ImageJ to quantify the microsclerotia amount using 8-bit grayscale transformation and set background threshold at 100, and then the particles were analyzed with the default settings for size (pixel^2^) and circularity. The quantification data were analyzed in *R* environment version 4.0. Because the data fulfilled the assumption of normality and homoscedasticity based on the Shapiro-Wilk test and Breusch-Pagan test (*P > *0.05), the data were analyzed using analysis of variance (ANOVA) and the Tukey honestly significant difference (HSD) test for mean separation.

In order to stain for ${\text{O}}_{2}^{-}$ and H_2_O_2_, NBT (BioShop) was prepared in 70% dimethyl sulfoxide and 5× PBS buffer (51), while the DAB (AK Scientific Inc., Union City, CA) was prepared in double-distilled water (ddH_2_O) via gradual acidification by 1 N HCl until the DAB powder was dissolved. The working concentration of both NBT and DAB was 0.05%, and the staining solution was passed through a 0.22-μm filter disk before storing at 4°C (48).

### ${\text{O}}_{2}^{-}$ and H_2_O_2_ stimulation assay and staining.

The SOD1 inhibitor sodium diethyldithiocarbamate trihydrate (DETC) (Alfa Aesar, Thermo Fisher Scientific, Ward Hill, MA), and 30% vol H_2_O_2_ (PanReac AppliChem) were freshly prepared in the 9-cm petri dish PDA plates to minimize the reduction of oxidants, and the assay was conducted as described for the antioxidants. The microsclerotia formation was photographed and quantified as mentioned in the antioxidant assay. Two photos were taken from each plate, and all conditions (DETC, H_2_O_2_, and control) were repeated three times with three biological replicates/plates in each repeat. The data were checked for the assumption of normality and homoscedasticity (*P > *0.05) before ANOVA and Tukey HSD test.

### Data availability.

The RNA-Seq raw data were deposited in the NCBI BioProject PRJNA752015.
